# Supplementary material for: Comparative Chloroplast Genomes of Six Magnoliaceae Species Provide New Insights into Intergeneric Relationships and Phylogeny
Source: Biology (Basel). 2022 Aug 28;11(9):1279. doi: 10.3390/biology11091279 (PMC9495354; doi:10.3390/biology11091279)
Supplement: Supplementary file 1 [file biology-11-01279-s001.zip › biology-1849287-supplementary.pdf]

Table S1 Chloroplast genomes of 32 species for phylogenetic analysis

Number	Latin name	Genbank number	genus
1	*Alcimandra cathcartii*	JX280390	*Alcimandra*
2	*Yulania amoena*	MN990623	*Yulania*
3	*Yulania biondii*	MN990596	*Yulania*
4	*Yulania cylindrica*	MN990617	*Yulania*
5	*Yulania denudata*	MN990618	*Yulania*
6	*Yulania liliiflora*	MN990588	*Yulania*
7	*Paramichelia baillonii*	MK782763	*Paramichelia*
8	*Tsoongiodendron odorum*	NC023239	*Tsoongiodendron*
9	*Liriodendron chinense*	KU170583	*Liriodendron*
10	*Liriodendron tulipifera*	MN990625	*Liriodendron*
11	*Manglietia aromatica*	MF990561	*Manglietia*
12	*Manglietia chevalieri*	MN990616	*Manglietia*
13	*Manglietia conifera*	MF990563	*Manglietia*
14	*Manglietia dandyi*	MF990567	*Manglietia*
15	*Manglietia duclouxii*	MF990564	*Manglietia*
16	*Manglietia glaucifolia*	MF990565	*Manglietia*
17	*Manglietia hainanensis*	MN306583	*Manglietia*
18	*Manglietia obovalifolia*	MZ597871	*Manglietia*
19	*Michelia lacei*	MW470924	*Michelia*
20	*Michelia maudiae*	MK631950	*Michelia*
21	*Pachylarnax sinica*	MN990584	*Pachylarnax*
22	*Parakmeria nitida*	MN990640	*Parakmeria*
23	*Parakmeria yunnanensis*	NC024545	*Parakmeria*
24	*Illicium henryi*	KY085910	*Illicium*
25	*Illicium verum*	KY085896	*Illicium*
26	*Kadsura heteroclita*	MN823698	*Kadsura*
27	*Kadsura longipedunculata*	MH535482	*Kadsura*
28	*Schisandra chinensis*	KU362793	*Schisandra*
29	*Schisandra sphenanthera*	KX638480	*Schisandra*
30	*Talauma hodgsonii*	MZ597871	*Talauma*
31	*Woonyoungia septentrionalis*	HM775382	*Woonyoungia*
32	*Arabidopsis thaliana*	JX280392	*Arabidopsis*

Table S2 Chloroplast genome-encoded gene types and functional classification for six species of the family Magnoliaceae

Category	Group	Genes
Photosynthesis	Subunits of photosystem I	psaA, psaB, psaC, pasI, pasJ
	Subunits of photosystem II	psbA, psbB, psbC, psbD, psbE, psbF, psbH, psbI, psbJ, psbK, psbL, psbM, psbN, psbT, psbZ,
	Subunits of NADH dehydrogenase	ndhA*, ndhB(×2)*, ndhC, ndhD, ndhE, ndhF,,ndhG,ndhH,ndhI,ndhJ, ndhK
	Subunits of cytochrome b/f complex	petA, petB*, petD, petG, petL, petN
	Subunits of ATP synthase	atpA, atpB, atpE, atpF*, atpH, atpI
	Large subunit of Rubisco	rbcL
Translation related genes	Large subunits of ribosome	rpl2(×2)*, rpl14, rpl16*, rpl20, rpl23 (×2), rpI32, rpl33, rpl36
	Small subunits of ribosome	rps2, rps3, rps4, rps7(×2), rps8, rps 11, rps12(×2)**,rps14,rps15,rps16, rps18, rps19
	DNA-dependent RNA polymerase	rpoA ,rpoB, rpoC1*, rpoC2,
	Ribosomal RNAs	rrn4.5(×2),rrn5(×2),rrn16(×2),rrn23(×2)
	Transfer RNAs	trnA-UGC(×2),trnC-GCA,trnD-GUC, trnE-UUC,trnF-GAA,trnFM-CAU, trnG-GCC, trnG-UCC*,trnH-GUG, trnI-CAU(×2),trnI-GAU(×2) *,trnK-UUU*,trnL-CAA(×2),rnL-UAA*, trnL-UAG,trnM-CAU,trnN-GUU(×2), trnP-UGG,trnQ-UUG,trnR-ACG(×2), trnR-UCU,trnS-GCU,trnS-GGA,trnS-UGA,trnT-GGU,trnT-UGU,trnV-GAC (×2),trnV-UAC,trnW-CCA,trnY-GUA
Other genes	Maturase	matK
	Protease	clpP**
	Envelope membrane protein	cemA
	Acetyl-CoA_carboxylase	accD
	C-type_cytochrome_synthesis_gene	ccsA
	Pseudogene	infA
	Genes of unknown function	ycf1, ycf2 (×2), ycf3**, ycf4, ycf15 (×2)

Note：(×2) indicates that the gene has two copies, * and ** indicate genes containing one/ two introns.

Table S3 Simple sequence repeats in the six chloroplast genomes of the family Magnoliaceae.

SSRs	*M. crassipes*	*M. grandis*	*M. hookeri*	*M. Ventii*	*Y. praecocissima*	*Y. soulangeana*
A/T	27	30	30	27	29	29
C/G	3	2	4	3	2	2
AG/CT	2	2	2	2	3	3
AT/AT	6	6	6	6	5	5
AAG/CTT	2	2	2	2	1	1
AAT/ATT	2	2	2	2	2	2
AATC/AATG	1	1	1	1	1	1
AAAT/ATTT	4	4	4	4	4	4
AATT/AATT	1	1	1	1	1	1
AATG/ATTC	2	2	2	2	1	1
ACAT/ATGT	1	1	1	1	1	1
AATACT/AGTATT	2	2	2	2	2	2
total	53	55	57	53	53	53
